# Measuring social norms related to child marriage among married women and men in Niger

**DOI:** 10.1371/journal.pone.0307595

**Published:** 2024-07-26

**Authors:** Pooja Sripad, Jessie Pinchoff, Chaibou Dadi, Leanne Dougherty

**Affiliations:** 1 Population Council, Washington, DC, United States of America; 2 Conception Etudes Suivi Evaluation Appuis Formation, Niamey, Niger; University of Salamanca, SPAIN

## Abstract

**Background:**

Child marriage remains prevalent in the Sahel region. Pervasive norms regarding child marriage, and sexual behavior persist. We explored norms from multiple perspectives to strengthen interventions aimed at delaying age at marriage.

**Methods:**

This study analyzed a cross-sectional household survey conducted in Niger in 2022 with women aged 15–49 (n = 2,726) and a subset of their male household members aged 15–59 (n = 1,136). Separate logistic regression models assessed factors associated with three descriptive (e.g., perception of what others do) and injunctive (e.g., perception of a group’s approval or disapproval) normative outcomes related to practices that support marriage as soon as a girl reaches puberty and beliefs related to premarital sex.

**Results:**

Our study found a greater proportion of men described early marriage as protective from the fear of socially induced ruined marital prospects for women (70% vs. 64%), while women expressed its protection from being harassed (62% compared to 42%). The injunctive norm outcome that “my neighbors think that one should marry off one’s daughter as soon as she reaches puberty” was significantly associated with the belief that child marriage was protective for females among women (OR = 4.49; 95% CI 3.13. 5.50) and men (OR = 8.21; 95% CI 5.88, 11.45).

**Conclusions:**

Programs addressing child marriage should consider both male and female perspectives to address differences and foster an environment where communities and families shift norms to delay early marriage.

## Introduction

Despite the global community’s commitment outlined in the Sustainable Development Goals to end child marriage, data from 34 sub-Saharan Africa countries between 2008 and 2017 found that nearly 54% of women aged 20–24 years were married before age 18 with the most prominent countries in West Africa where several countries have a prevalence of more than 70% [1]. While evidence on the causality between child marriage and health outcomes is limited [2], several studies have found that women who experience child marriage compared to women married as adults (i.e., 18 or older) were less likely to use modern contraception [1], initiate antenatal care (ANC), or attend four or more ANC visits during pregnancy [3] and were less likely to access a skilled birth attendant or use postnatal care following delivery [4]. They were also more vulnerable to reproductive coercion, and sexual intimate partner violence [5–7]. A study conducted in Niger and Ethiopia found that marriage at age 15 or earlier was negatively associated with psychological well-being [8]. Previous studies have also found that girls who marry early are more likely to drop out of school, have lower earning potential and contribute less to household economic and health care decisions [9]. This may be because males expect obedience and adolescent girls have a lower expectation to be involved in decisions [10].

Previous studies have considered the drivers of child marriage and have identified factors at the individual, household and community levels [11–15]. Psaki et al developed a framework that describes the drivers of child marriage and proposes that social norms and attitudes as well as poverty and economic factors form part of a larger social context in which decisions about marriage are made and precede other drivers of child marriage including lack of agency, lack of opportunities (i.e., education and work) and fear of girls’ sexuality and pregnancy [16]. The link between social norms and child marriage has increasingly been documented [16, 17], with a growing understanding that social norms influence communities and family members, perpetuating the practice of child marriage [18]. Social norms, broadly speaking, reflect collective practices that arise and are influenced by what other people do and by what other people think should be done [19]. According to Bicchieri et al, as opposed to customs and moral rules, social norms reflect the collective practices sustained by empirical and normative expectations around a practice and preferences conditional on these expectations (e.g., most people conforming to or believing they ought to conform to a norm). Social norms can be deconstructed as perceptions of what others within one’s group does (descriptive norms), perceptions of a group’s approval or disapproval of one’s non-conformity (injunctive norms), and interactions between the two [19, 20]. Social norms promoting child marriage relate to feared love affairs constraining marriage prospects, discouraged pre-marital sexual activity, and a fear of poverty [21]. The prevalence of and influences on these norms, when deconstructed as descriptive and injunctive norms, can be assessed contextually using quantitative data.

The United States Agency for International Development’s (USAID) Resilience in the Sahel Enhanced (RISE) II project targets chronically vulnerable populations through integrated programming to improve health behaviors and outcomes including reducing rates of child marriage in Niger [22]. Niger has one of the highest rates of child marriage, with approximately 77% of women between the ages of 20 and 49 marrying before the age of 18 and 30% marrying before the age of 15 [23]. The Niger Civil Code sets the minimum age of marriage at 18 years for boys and 15 years for girls and the majority of unions take place under customary law where there is limited enforcement or opportunities for legal action [24]. Moreover, social norms around child marriage in Niger are intertwined with broader vulnerabilities including poverty and a lack of opportunities for girls outside of marriage [16]. An adolescent girl’s family plays a dominant role in determining who she marries and when. According to the 2012 Niger Demographic and Health Survey, two-thirds of all married women of reproductive age stated that their father decided who she married and approximately half said that a mother should give her daughter for marriage before the age of 18 [23]. Previous qualitative research from Niger finds that girls and their families make marital decisions within a context that elevates parental consent and community approval, and is constrained by limited economic and educational prospects, the gendered distribution of labor, and social norms that promote a narrow ‘window of opportunity’ for marriage [25]. The rise in violent extremism in the Sahel [26] further complicates the situation as conflict can amplify pre-existing drivers of child marriage particularly related to conservative norms around family honor [27]. Quantitative research from Niger reported that village level norms related to marital choice (e.g., who had the greatest say about arranging your marriage to your husband?) were associated with younger age of girls at marriage [28]. Data further suggest that among women of reproductive age, the reasons for why a mother would give her daughter for marriage below the age of 18 were primarily related to avoiding pregnancy outside of marriage (25%) and avoiding sexual promiscuity (19%) [23]. These findings suggest that there may be strong social norms related to the importance of an adolescent girl’s chastity and that families may believe premarital sex would ruin the reputation of both the daughter and parents.

**Fig 1:**
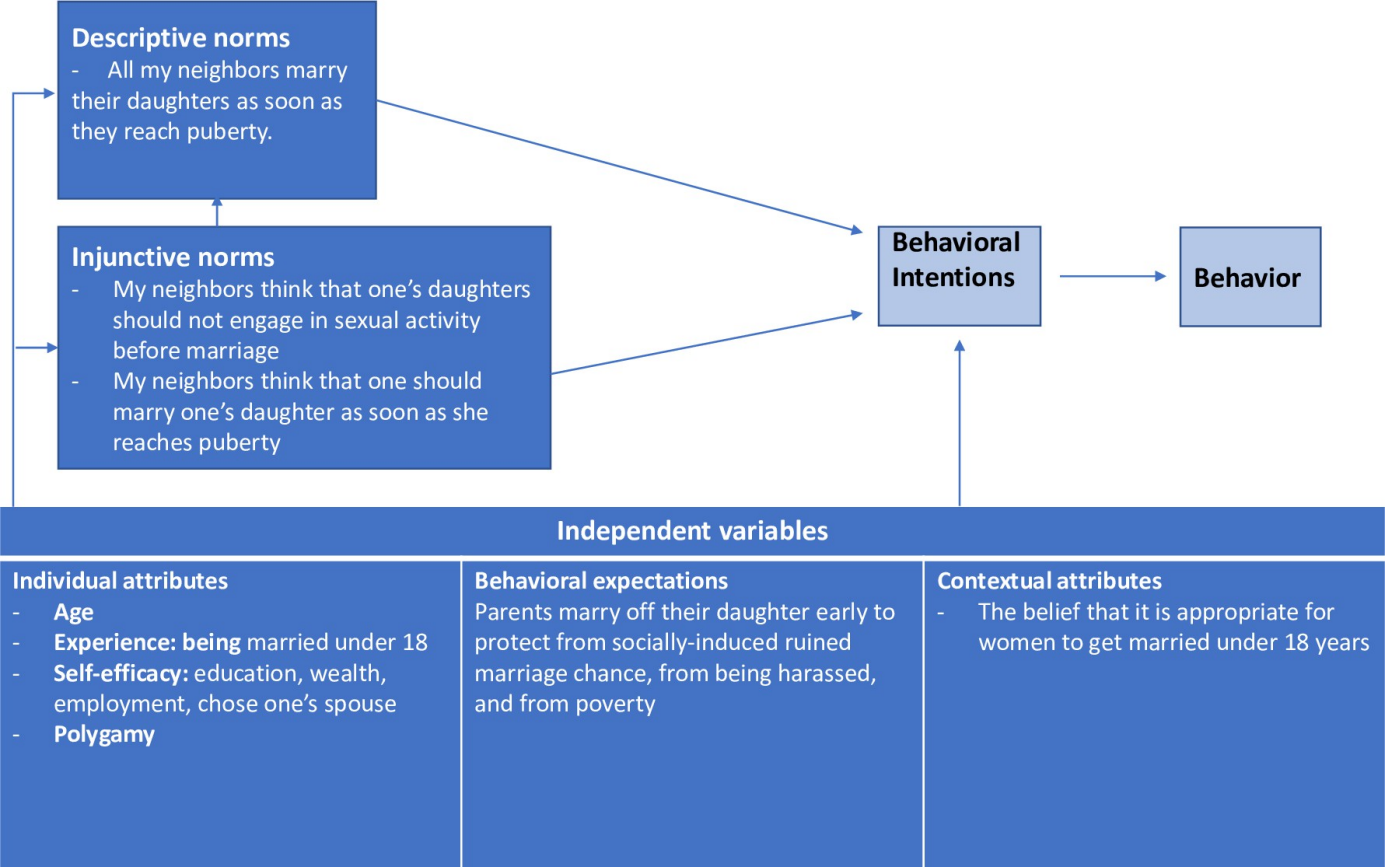
Conceptual framework.

The purpose of this study was to describe social norms around child marriage and how related individual, behavioral, and contextual factors influence these norms in RISE II program areas. Understanding social normative influences on child marriage can both inform RISE II community engagement programming that address social norms by sensitizing parents and community members on the risks of child marriage as well as inform other child marriage prevention and identification efforts more broadly. Drawing on the Chung and Rimal (2016) revised normative influences framework developed from a review of conceptual and empirically based social norms theories, we explored child marriage descriptive and injunctive norms and their association with behavioral, individual, and contextual attributes as precursors of behavioral intent and ultimately behavior [20]. In Fig 1, variables considered in our analysis are in dark boxes and are precursors to the ultimate outcomes of behavioral intentions (e.g., intent to marry daughter/girl before 18) and behavior (e.g., girl is married before the age of 18) reflected in the light boxes. Behavioral expectations refer to if the individual believes the behavior will result in a positive or negative outcome. Individual attributes include items such as age and past experience (e.g., experience being married under 18). Contextual attributes refer to items such as beliefs (e.g., belief that it is appropriate to get married under 18) and injunctive norms (e.g. that neighbors think one’s daughters should not engage in sexual activity before marriage) which can also be associated with descriptive norms. Unlike prior research, this study explored patterns of social norms around child marriage from both women’s and men’s perspectives, with an emphasis on norms related to fear of girls’ sexual behaviors and pregnancy.

## Materials and methods

**Fig 2:**
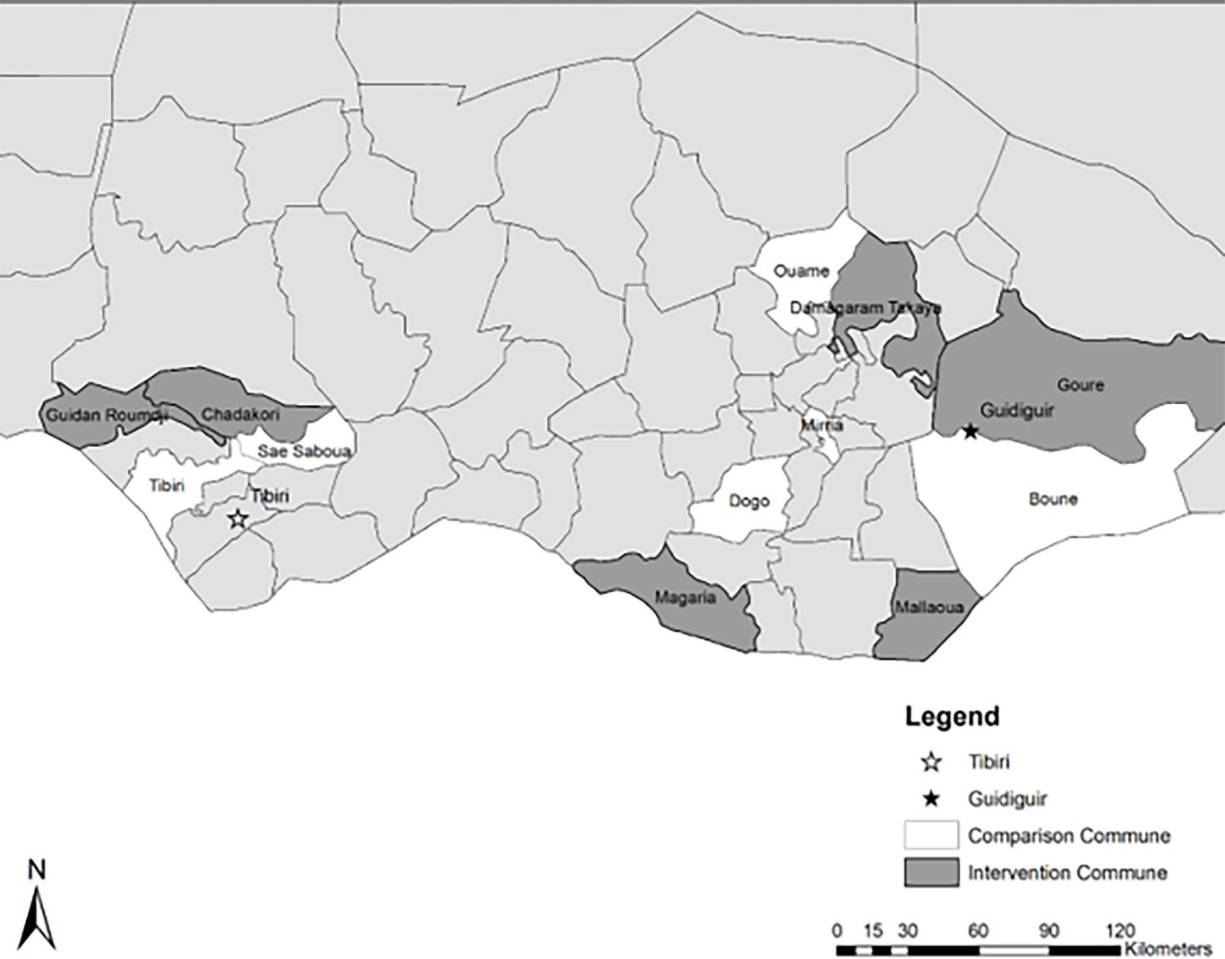
Map of Niger and RISE II study areas.

This study drew from USAID’s Breakthrough RESEARCH integrated social and behavior change (SBC) evaluation of the RISE II program and used a cross-sectional household survey collected in April 2022 in Niger’s Maradi and Zinder regions around the midpoint of the intervention, as shown in Fig 2. The rationale for using the cross-sectional midline household survey was because it provided the most up to date measures related to child marriage social norms in the study area. Since the midline is conducted early in the implementation, we adjust for commune in the model to acknowledge programming was assigned by commune, but we are not evaluating the effectiveness of the interventions at this time. We are reporting a cross-sectional analysis of norms for respondents from these communities and adjust for potential recent program exposure, however, programming was 1) not exclusively about child marriage; 2) the programming modality and intensity varied by commune; and 3) has been ongoing in the region for decades. The survey reached approximately 2,700 married women and 1,300 married men who lived in the same household.

### Study site and program

The RISE II integrated SBC program began in 2019 and was implemented at the community level through the Resilience Food Security Assistance (RFSA) partners in the Maradi and Zinder regions of Niger. RFSAs used a variety of SBC approaches, including community engagement and interpersonal communication (IPC) through peer group activities and radio to address a range of health and development outcomes including child marriage [29]. Maradi and Zinder are densely populated, agriculturally based regions in southern Niger compared to the northern part of the country which is largely desert and nomadic and are less affected by terrorists operating on the Mali and Burkina Faso borders and northern parts of the country. The median age of marriage for women is 15 years old [23].

### Sampling

We applied a three-stage stratified sampling procedure. In the first stage, we randomly selected six intervention communes from the 18 intervention communes and six comparison communes (four in Zinder and two in Maradi region per intervention and comparison areas). In the second stage, we listed all enumeration areas (EA) identified in the 2012 General Census by commune in each of the randomly selected communes. We then used probability proportion to size to select EAs per commune starting at a random point and then systematically selecting areas using a fixed sampling interval. In total, we sampled 40 EAs for each group, stratified by commune. In the third stage, we enumerated all households in each of the randomly selected EAs with eligible women (married women between 15 and 49 years of age and married men between 15 and 59 years of age). From households with eligible study participants, we randomly selected 34 households per EA to account for a 10% non-response rate and interviewed approximately 30 eligible women and 15 eligible men. A participant was randomly chosen (by flip of a coin) if the household had more than one eligible participant. A larger sample of women was required to ensure adequate power for specific subpopulation study outcomes. We did not match husbands with females interviewed and did not register the proportion of male household members who were husbands of female respondents. Response rates were nearly 100% and we arrived at a final sample size of 2,726 women and 1,136 men for analysis. This sample size is based on a minimum detectable difference for evaluation purposes of 6–11 percentage points in the priority indicators (e.g., presence of a handwashing station at the household) between study groups, with 80% power to detect a difference and α = 0.05. Background characteristics between intervention and comparison areas were overall not statistically significant different at baseline [30].

### Measures

The study draws from the RISE II integrated SBC study instruments which were designed to quantitatively assess changes in ideational factors (including social and gender norms), attitudes, health intentions, decision making and behaviors for a range of reproductive, maternal and child health outcomes. Table 1 provides a description of variables used in the analysis.

**Table 1:** Description of variables used in analysis.

Category	Variable	Construction	Type
Individual attribute	Age	Continuous item in years	Explanatory
Individual attribute	Married at age ≤ 18 years	Binary variable, age at marriage ≤ 18 years or older (women only)	Explanatory
Individual attribute	Wealth	Categorical variable, household wealth tercile using methods from the Equity Tool [31]	Explanatory
Individual attribute	Education	Binary variable, defined as no education or any formal schooling	Explanatory
Individual attribute	Currently working	Binary variable, worked in the last 12 months or not	Explanatory
Individual attribute	Polygamous marriage	Binary variable, married/monogamous, or married/polygamous, based on the questions “Are you currently married, living with a man like you are married?” and “Does your (husband/partner) have other wives?”	Explanatory
Individual attribute	Involvement in marriage decision	Binary variable, “Chose own spouse” vs family chose	Explanatory
Behavioral expectations	Summary of three variables regarding beliefs that child marriage is protective (Child marriage is defined as any formal or informal union between a child under the age of 18 and an adult or another child)	Continuous variable, summing agreement with three questions: Parents marry off daughters early because they are afraid otherwise, she might have love affairs that ruin her chance of a decent marriage Child marriage protects a girl from being harassed Child marriage protects a girl from poverty	Explanatory
Contextual attribute	Moral rules regarding appropriateness of early marriage	Binary variable, created from “age it is appropriate to get married”;≤18 years vs older (women only)	Explanatory
Descriptive norms	“All my neighbors marry off their daughters as soon as they reach puberty”	Binary variable, “All my neighbors marry off their daughters as soon as they reach puberty” agree or disagree/don’t know	Outcome
Injunctive norms	“My neighbors think that one’s daughters should not engage in sexual activity before marriage”	Binary variable, “My neighbors think that one’s daughters should not engage in sexual activity before marriage” agree vs disagree/don’t know	Outcome/ Explanatory
Injunctive norms	“My neighbors think that one should marry off one’s daughter as soon as she reaches puberty”	Binary variable, “My neighbors think that one should marry off one’s daughter as soon as she reaches puberty” agree vs disagree/don’t know	Outcome/ Explanatory

#### Outcome variables

The three main child marriage norms outcomes explored were: 1) agreeing with the descriptive norm “all my neighbors marry off their daughters as soon as they reach puberty”; 2) agreeing with the injunctive norm “My neighbors think that one’s daughters should not engage in sexual activity before marriage”; and 3) agreeing with the injunctive norm “My neighbors think that one should marry off one’s daughter as soon as she reaches puberty”. Social norms questions were adapted from Bicchieri’s previous work focused on a social norms perspective on child marriage [19].

#### Independent variables

Individual attributes included age, whether the respondent was married at 18 years of age or less and whether they were involved in the marriage decision, wealth status, educational attainment, currently working, and whether the marriage was polygamous or monogamous. The behavioral expectations measure was constructed based on three questions as described in Table 1. Using a Cronbach alpha test, we determined that the behavioral expectation items were correlated and therefore constructed a score by summing agreement with the three questions. The contextual attribute assessed moral rules regarding appropriateness of early marriage and an injunctive norm.

### Data collection procedures

Conception-Etudes-Suivi-Evaluation-Formation (CESAF), a Niger based research firm, was responsible for data collection. CESAF recruited interviewers locally with previous experience with household surveys and trained them on the objectives of the research and obtaining written informed consent (or verbal consent if the participant was unable to write). Interviewers administered the survey in Hausa to the study participants using mobile phones through the SurveyCTO mobile data collection application. Interviews took place in-person at the participant’s home in an outdoor and private location and interviews lasted approximately 30–40 minutes.

### Statistical analysis

Guided by our conceptual framework, we first conducted a descriptive analysis followed by multivariable logistic regression models to explore the extent to which behavioral expectations, individual attributes, and contextual attributes were associated with child marriage norms. Given the similarity in variable construction between descriptive and injunctive norms, we ran correlation tests. We found that the descriptive norm, “all my neighbors marry off their daughters as soon as they reach puberty” was highly correlated with the injunctive norm “My neighbors think that one should marry off one’s daughter as soon as she reaches puberty”. However, neither of these outcomes was associated with the third injunctive norm “My neighbors think that one’s daughters should not engage in sexual activity before marriage”. As a result, we only included the non-correlated variable in each model. Models were run stratified by gender. The belief that it was appropriate for women to marry before the age of 18 years variable and whether the woman was married before the age of 18 was only assessed among women. We adjusted for standard errors by strata and commune to account for the sampling approach and study area. Because of the widespread vulnerabilities in the region, there are overlapping development programs operating in the RISE II study areas. As such, individuals interviewed for this study were exposed in both the intervention and comparison areas to child marriage prevention messages. At the time data were collected, we found exposure to messages varied primarily by study region than by study group as described in the RISE II midline report and therefore focused our analysis on adjusting by study area [32]. Data cleaning and analyses were completed using Stata software version 16 [33].

#### Ethics

This study was approved by the Population Council Institutional Review Board (New York, NY, USA, protocol #934) and approved locally in Niger by The Ministry of Public Health National Ethics Committee for Health Research in Niger (No. 017/2020/CNERS). The informed consent was read to the participants by a research assistant trained in ethical human subjects research. The consent form used lay language and the participants were given the opportunity to ask questions about the study before giving consent. Study participants provided informed consent by marking agreement (in the form of an X) without using their signature and were informed that they could withdraw at any point without experiencing any consequence. This procedure is prevalent in similar study settings and allows for the inclusion of illiterate individuals or those without defined signatures. Women interviewed between the ages of 15–17 were emancipated minors and therefore parental consent was not required. In addition, all methods were carried out in accordance with relevant guidelines and regulations and with the 1964 Helsinki declaration and its later amendments or comparable ethical standards.

## Results

### Background characteristics of study participants

Our samples consisted of 2,726 women and 1,136 men with approximately two-thirds living in the Zinder region as shown in Table 2. Women were younger with an average age of 32 years compared to men with an average age 43 years. Less than 20% of women completed any schooling (14%) nor worked (18%), while the majority were married under the age of 18 years (80%). Many women reported being able to choose their spouse (41%), and a little over a third were in polygamous marriages (38%). Men similarly held low levels of education, though the majority (78%) were currently employed. About a third of men reported being in polygamous marriages (30%) and 39% chose their spouse.

**Table 2:** Descriptive characteristics of sample.

	Women N = 2,726		Men N = 1,136		p-value
	N	% (CI)	N	% (CI)	
Region					0.286
Maradi	956	35% (34.0–36.0)	411	36% (33.8–38.4)	
Zinder	1776	65% (64.0–66.0)	725	64% (61.6–66.2)	
*Individual attributes*					
Age (mean, SD)	31.7 (6.7)		43.1 (10.5)		0.000
Wealth tercile					0.002
Lowest	914	34% (28.2–39.2)	347	31% (25.0–36.5)	
Middle	908	33% (29.9–36.8)	367	32% (28.3–36.6)	
Highest	910	33% (27.9–39.0)	422	37% (31.2–43.7)	
Finished any schooling	376	14% (11.7–16.5)	213	19% (14.5–24.1)	0.003
Worked in last 12 months (currently working)	490	18% (16.1–19.7)	884	78% (75.3–90.3)	0.000
Married at ≤18 years	2,026	80%	NA	NA	NA
Polygamous marriage	1,007	38% (33.9–39.3)	341	30% (26.5–32.9)	0.000
Chose own spouse (vs. family, others)	1,110	41% (38.7–42.8)	300	39% (35.6–42.3)	0.103
*Behavioral expectations*					
Marrying before the age of 18 protects a female from poverty + harassment + socially induced, ruined marriage prospects (composite score; values 0–3)	1.53 (1.09)		1.39 (1.09)		0.000
Fear that she might have love affairs and ruin her chance of a decent marriage	1,738	64% (62.0–67.0)	788	70% (66.8–73.5)	0.003
Child marriage protects a girl from being harassed	1,691	62% (60.0–65.2)	470	42% (38.5–45.6)	0.000
Child marriage protects a girl from poverty	720	27% (24.6–28.9)	305	27% (24.6–30.2)	0.742
*Contextual attributes*					
Think ≤18 is appropriate age to get married	2,264	83%			NA
My neighbors marry off their daughters as soon as they reach puberty (*Descriptive norm*)	1,385	54% (51.0–56.5)	699	63% (60.1–66.4)	0.000
My neighbors think that one’s daughters should not engage in sexual activity before marriage (*Injunctive norm)*	1,634	63% (61.0–65.1)	851	77% (74.0–80.4)	0.000
My neighbors think that one should marry off one’s daughter off as soon as she reaches puberty (*Injunctive norm)*	1,382	54% (50.6–56.7)	695	63% (59.9–66.2)	0.000

Behavioral expectations that early marriage is protective for women were similar across women and men. However, a greater proportion of men described early marriage as protective from the fear of socially induced ruined marital prospects for women (70% vs. 64%), while women expressed its protection from being harassed (62% compared to 42%); about a quarter of men and women expressed child marriage as a means of protection from poverty (27%). The contextual belief that it is appropriate to get married under 18 years of age was 83% among women. Men reported statistically significant higher levels of agreement with the descriptive and injunctive norms compared to women (63% vs. 54%; 77% vs. 63%; 63% vs. 54%).

### Descriptive norm and injunctive norms among women

#### My neighbors marry off their daughters as soon as they reach puberty (descriptive norm)

We first considered the individual attributes associated with the descriptive norm. Among women in wealthier terciles and those that reported having been married early themselves had lower odds of agreeing with the descriptive norm “All my neighbors marry off their daughters as soon as they reach puberty” (Table 3). While women who were in a polygamous marriage, chose one’s spouse, and were employed had higher odds of agreeing with this descriptive norm. Age and education were not associated with the perception of one’s neighbors marrying off their daughters as soon as they reach puberty.

**Table 3:** Factors associated with descriptive child marriage norm and injunctive child marriage norms among women in Zinder and Maradi, Niger.

	My neighbors marry off their daughters as soon as they reach puberty (N = 2,259) (*Descriptive Norm*)	My neighbors think that one’s daughters should not engage in sexual activity before marriage (N = 2,264) (*Injunctive norm*)	My neighbors think that one should marry off one’s daughter as soon as she reaches puberty (N = 2,264) (*Injunctive norm*)
VARIABLES	Adjusted ORs	Adjusted ORs	Adjusted ORs
***Individual attributes***			
Age (in years)	1.00 (0.98–1.01)	0.99 (0.97–1.01)	1.00 (0.98–1.02)
Wealth tercile = 1, poorest (reference category (REF))	REF	REF	REF
Wealth tercile = 2, middle	0.62* (0.43–0.91)	1.63** (1.24–2.15)	0.62* (0.42–0.91)
Wealth tercile = 3, richest	0.61* (0.41–0.89)	1.54** (1.21–1.95)	0.62* (0.42–0.93)
Any formal schooling (vs. none)	0.86 (0.62–1.19)	1.35* (1.02–1.79)	0.76 (0.55–1.06)
Currently working (vs. not)	2.02** (1.49–2.73)	2.19** (1.60–2.98)	1.80** (1.34–2.42)

Polygamous marriage (vs. monogamous)	1.35* (1.07–1.71)	1.14 (0.90–1.44)	1.33* (1.02–1.72)
Was married at ≤age 18 years (vs. >18 years)	0.69* (0.53–0.92)	1.38* (1.08–1.75)	0.70* (0.53–0.92)
Chose own spouse (vs. family, other chose)	3.72** (2.84–4.86)	0.56** (0.45–0.70)	4.15** (3.13–5.50)
***Behavioral expectations***			
Marrying before the age of 18 protects females^A^ (yes vs. no)	4.84** (4.07–5.74)	1.50** (1.33–1.69)	4.49** (3.77–5.34)
***Contextual attributes***			
Thinks ≤18 yr is appropriate age to marry (yes vs. no)	0.73 (0.46–1.15)	0.79 (0.57–1.10)	0.86 (0.51–1.45)
My neighbors think that one should marry off one’s daughter as soon as she reaches puberty (yes vs no)	NA	3.27** (2.54–4.21)	NA
My neighbors think that one’s daughters should not engage in sexual activity before marriage (yes vs no)	2.56** (2.00–3.29)	NA	3.33** (2.56–4.33)
Constant	0.09** (0.04–0.20)	0.55 (0.29–1.01)	0.07** (0.03–0.15)

** p<0.01

* p<0.05; confidence interval in parentheses

^A^ from poverty, harassment and socially induced, ruined marriage prospects

Next, we considered the behavioral expectations attribute. Women who reported believing that child marriage was protective for females were significantly more likely to agree with the descriptive norm (OR = 4.84; 95% CI 4.07, 5.74).

Finally, we examined the contextual attributes. The descriptive child marriage norm was significantly associated with the injunctive norm “that one’s daughters should not engage in sexual activity before marriage” (OR = 2.56; 95% CI 2.00, 3.29).

#### Engaging in sexual activity before marriage (injunctive norm)

When considering individual attributes, women who reported that they chose their spouse had lower odds of agreeing with the injunctive norm (“My neighbors think that one’s daughters should not engage in sexual activity before marriage”) (Table 3). Women in wealthier terciles, had any education, were working, or reported having been married early themselves had significantly higher odds of agreeing with the injunctive norm. Age and being in a polygamous marriage were not associated with the perception that one’s daughters should not engage in sexual activity before marriage.

The behavioral expectations attribute found that women who reported that child marriage was protective for females had an increased odds of agreeing with the injunctive norm related to premarital sex (OR = 1.50; 95% CI 1.33, 1.69).

In terms of contextual attributes, the injunctive norm related to premarital sex was also significantly associated with the other injunctive norm that one should marry off one’s daughter soon after puberty (OR = 3.27; 95% CI 2.54, 4.21).

#### Marry one’s daughter as soon as she reaches puberty (injunctive norm)

We considered the individual attributes associated with the injunctive norm related to marrying one’s daughter as soon as she reaches puberty. Among women, controlling for covariates, the injunctive norm promoting early marriage soon after puberty (“My neighbors think that one should marry off one’s daughter as soon as she reaches puberty”) was negatively associated with those in wealthier terciles and those who reported having been married early themselves. Conversely, women currently employed, in a polygamous marriage, reported they chose their own spouse (OR = 4.15; 95% CI 3.13, 5.34), had higher odds of agreeing with this injunctive norm. Age and education were not associated with the perception that one should marry off their daughters soon after reaching puberty.

For behavioral expectations, women that reported that child marriage was protective for females were positively associated with the perception that one should marry off their daughters soon after reaching puberty (OR = 4.49; 95% CI 3.13. 5.50).

For contextual attributes, the injunctive norm was also positively associated with the injunctive norm disapproving pre-marital sexual activity (OR = 3.33; 95% CI 2.56, 4.33).

### Descriptive norm and injunctive norms among men

#### My neighbors marry off their daughters as soon as they reach puberty (descriptive norm)

For men, we considered the individual attributes associated with the descriptive norm. Men in wealthier terciles, and who were working had significantly lower odds of agreeing with the descriptive child marriage norm (“All my neighbors marry off their daughters as soon as they reach puberty”) (Table 4). Age, education, being in a polygamous marriage, or choosing one’s spouse was not associated with the perception of ones’ neighbors marrying their daughters as soon as they reach puberty.

**Table 4:** Factors associated with descriptive norm and injunctive norms associations for men.

	My neighbors marry off their daughters as soon as they reach puberty (N = 1,065) (Descriptive Norm)	My neighbors think that one’s daughters should not engage in premarital sexual activity (N = 1,064) (Injunctive norm)	My neighbors think that one should marry off one’s daughter as soon as she reaches puberty (N = 1,064) (Injunctive norm)
VARIABLES	Adjusted ORs	Adjusted ORs	Adjusted ORs
***Individual attributes***			
Age (in years)	0.98	1.00	0.99
	(0.96–1.00)	(0.98–1.02)	(0.96–1.01)
Wealth tercile = 1, poorest (REF)	REF	REF	REF
Wealth tercile = 2, middle	0.55**	0.62	0.54**
	(0.36–0.86)	(0.34–1.15)	(0.35–0.85)
Wealth tercile = 3, richest	0.63*	0.27**	0.64*
	(0.41–0.99)	(0.13–0.57)	(0.39–1.03)
Any formal schooling (vs none)	1.04	1.38	1.19
	(0.55–1.99)	(0.75–2.52)	(0.59–2.41)
Currently working (vs not working)	0.42*	5.07**	0.47*
	(0.19–0.93)	(2.355–10.894)	(0.23–0.98)
Polygamous marriage (vs monogamous marriage)	1.17	4.43**	1.15
	(0.68–2.02)	(2.01–10.89)	(0.74–1.79)
Chose own spouse (vs family, other chose)	1.45	0.69	1.2
	(0.94–2.23)	(0.39–1.24)	(0.74–1.79)
***Behavioral expectations***			
Marrying before the age of 18 protects females^A^ (yes vs no)	8.39**	4.09**	8.21**
	(5.78–12.18)	(2.60–6.45)	(5.88–11.45)
***Contextual attributes***			
My neighbors think that one should marry one’s daughter as soon as she reaches puberty (yes vs no)	NA	9.99**	NA
		(5.52–18.08)	
My neighbors think that one’s daughters should not engage in sexual activity before marriage (yes vs no)	9.87**	NA	11.70**
	(4.36–22.34)		(5.51–24.84)
Constant	0.15*	0.17*	0.10*
	(0.03–0.66)	(0.049–0.57)	(0.02–0.45)

** p<0.01

* p<0.05

confidence interval in parentheses; ^A^ from poverty, harassment and socially induced, ruined marriage prospects

Regarding the behavioral expectations attribute, men who reported the belief that child marriage was protective for females (OR = 8.39; 95% CI 5.78, 12.18) had significantly higher odds of agreeing with the descriptive norm.

For the contextual attribute, the descriptive norm was associated with the injunctive norm that one’s daughters should not engage in sexual activity before marriage (OR = 9.87; 95% CI 4.36, 22.34).

#### Engaging in sexual activity before marriage (injunctive norm)

When considering individual attributes among men, controlling for covariates, the injunctive norm disapproving pre-marital sexual activity (“My neighbors think that one’s daughters should not engage in sexual activity before marriage”) was negatively and significantly associated with those in the wealthiest tercile (Table 4). Men that were in a polygamous marriage, or were working, had significantly higher odds of agreeing with the injunctive norm disapproving pre-marital sexual activity. Neither age nor education were associated with the perception that one’s daughters should not engage in sexual activity before marriage.

For the behavioral expectation attribute, men who expected that child marriage was protective for females had significantly higher odds of agreeing with the injunctive norm disapproving pre-marital sexual activity (OR = 4.10; 95% CI 2.60, 6.45).

Additionally for the contextual attribute, this injunctive norm was also associated with the other injunctive norm around early marriage soon after puberty (OR = 9.99; 95% CI 5.52, 18.08).

#### Marry off one’s daughter as soon as she reaches puberty (injunctive norm)

We considered the individual attributes associated with the injunctive norm related to marrying one’s daughter as soon as she reaches puberty. Among men, those in wealthier terciles or were working had significantly lower odds of agreeing with the injunctive norm promoting early marriage soon after puberty (“My neighbors think that one should marry off one’s daughter as soon as she reaches puberty”) (Table 4). Neither age, education, polygamy, nor choice of spouse were associated with the perception that one should marry off their daughters soon after reaching puberty.

For the behavioral expectations attribute, men that had the expectation that child marriage was protective for females (OR = 8.21; 95% CI 5.88, 11.45) had significantly higher odds of agreeing with the injunctive norm.

For the contextual attribute, the norm that “my neighbors think that one’s daughters should not engage in sexual activity before marriage” (OR = 11.70; 95% CI 5.51, 24.84) had significantly higher odds of agreeing with the injunctive norm.

## Discussion

Understanding social norms related to child marriage and their association with individual, behavioral, and contextual attributes among men and women can provide insights that support the development of more targeted SBC interventions that seek to influence communities and family members perpetuating the practice of child marriage. In this paper, we described social norms around child marriage and how related factors influence these norms in the RISE II program areas in Niger. Unlike prior studies that centered on women’s perspectives, we extended our understanding to include male viewpoints, particularly related to girls’ sexual behaviors as integral in shaping the social normative environment within which child marriage occurs.

Overall, we found that male and female perspectives around social norms—including associations of individual, behavioral, and contextual attributes with descriptive and injunctive norms—demonstrated similarities and differences. Differences may be related to individual attributes (i.e., wealth). Men reported higher agreement with descriptive and injunctive norms. Both men and women reported expectations that early marriage is protective for women. Although, women described its protective influence at higher levels—and there was variation within its components (while both saw it as somewhat protective from poverty, men saw marriage as socially protective from ruining martial chances due to pre-marital sex, while women saw it as protection from harassment). Given these divergent perspectives, future research should consider exploring women’s experience with harassment and how they see marriage as an opportunity for protection.

Broadly speaking for both men and women, agreement with descriptive and injunctive norms increased with behavioral attributes, particularly the expectations that early marriage protects women from physical and social harms such as ruined marriage prospects, harassment, and poverty. While agreement with the descriptive and injunctive norms around marrying one’s daughter as soon as she reached puberty was negatively associated with wealthier women and women who were married before the age of 18, the injunctive norm of disapproving women’s pre-marital sexual activity was positively associated with these individual attributes. Future research is needed to understand community perceptions of puberty and pre-marital sex. Interestingly, the strength of association between injunctive norms and with descriptive norms were more robust among men compared to women—suggesting that males may be more influenced by perceptions of what their neighbors think when it came to child marriage and female sexual behavior.

Choosing one’s spouse was associated with increased agreement with the descriptive and injunctive norm around ‘marriage soon after puberty for women’ and decreased agreement with the injunctive norm around disapproving pre-marital sex for men. Given the role that family members, particularly fathers, play in determining when a girl is married, more effort may be needed to understand intergenerational communication [11] and to increase awareness of the consequences of child marriage with adolescent girls’ families and their communities [34, 35]. Previous findings from a study in Ethiopia, India, Peru, and Vietnam found that girls who reported good parent-child communication and high parent-child relationship quality were less likely to marry early [36]. However, in Niger, parents reported a lack of knowledge and skills to initiate conversations related to sexual and reproductive health and face adverse barriers to open intergenerational communication [37]. In Senegal, an approach that aimed to empower the elder community of women to improve intergenerational communication related to child marriage including advocating on their behalf resulted in adolescent girls feeling improved agency and norms were shifting to support delayed marriage [38].

While education and employment have previously been shown to be a protective factor in the prevention of child marriage [39, 40] including in Niger [41], age and education were not associated with child marriage norms for women in the adjusted models in our study. However, wealth may have a mitigating effect for men and women. Previous studies have found that interventions that aim to increase education or economic opportunities among young girls have had a positive effect in reducing the practice of child marriage [42–45]. The effectiveness of these interventions on shifting related social norms remains unclear and warrants further exploration.

### Limitations

Since the data for this study were drawn from a broader evaluation of which child marriage norms was not the primary focus, the full pathway of norms relationships with attributes to behavioral intent was not represented in the questionnaire design. The limited set of questions available for analysis also did not address gender norms as an important driver of child marriage [46, 47], future child marriage intentions and behaviors for a sister or a daughter (given our sample was mostly married) as well as details on factors influencing males’ choices and attitudes related to marriage which would be useful to investigate in future studies. While we were able to assess the appropriateness of marriage before 18 years in the woman’s questionnaire, the question was a bit problematic in the men’s questionnaire—because men responded about their own sex instead of whether ‘it is appropriate for women to get marriage under 18 years’—asking the latter may have elicited a more congruent belief with respect to girl’s child marriage. The cross-sectional study design did not enable a cause-and-effect relationship to be established which would have been feasible with a longitudinal study. Finally, to address specific cultural nuances, future research should consider conducting qualitative research to identify local practices and attitudes to ensure social norm interventions reflect an understanding of the context [48]. Individual and/or collective in-depth narratives on child marriage and female sexual behaviors in Maradi and Zinder would enable us to triangulate, clarify, and reinforce our findings around women’s and men’s behavioral expectations and contextual attribute influences on descriptive and injunctive norms through illustrative examples.

### Conclusion

Our study found that women who were able to choose their husband and were wealthier were less likely to agree with social norms related to fear of pregnancy and sexual activity among young girls. Furthermore, we found strong associations among men and women related to protective expectations of child marriage whereby respondents who supported harmful social norms related to fear of pregnancy and sexual activity among young girls were more likely to believe that child marriage protected from poverty, harassment and socially induced ruined marriage prospects among young girls. In addition, we found that men were more affected by injunctive normative influence, or others’ perceptions of child marriage and restricting female’s sexual behaviors—and may require a targeted norms intervention. Future programs should consider how to address social norms related to fear of pregnancy and sexuality among young girls when planning interventions and seek to foster an environment where communities and families engage in intergenerational communication to empower young girls and shift attitudes and subsequent norms to delay early marriage.

## Abbreviations

ANC: Antenatal care
EA: Enumeration area
IPC: Interpersonal communication
OR: Odds ratio
RFSA: Resilience Food Security Activity
RISE: Resilience in the Sahel Enhanced
SBC: Social and Behavior Change
USAID: United States Agency for International Development
